# Decreased Prefrontal Lobe Interhemispheric Functional Connectivity in Adolescents with Internet Gaming Disorder: A Primary Study Using Resting-State fMRI

**DOI:** 10.1371/journal.pone.0118733

**Published:** 2015-03-04

**Authors:** Yao Wang, Yan Yin, Ya-wen Sun, Yan Zhou, Xue Chen, Wei-na Ding, Wei Wang, Wei Li, Jian-rong Xu, Ya-song Du

**Affiliations:** 1 Department of Radiology, Ren Ji Hospital, School of Medicine, Shanghai Jiao Tong University, Shanghai, P.R. China; 2 Department of Child & Adolescent Psychiatry Shanghai Mental Health Center, Shanghai Jiao Tong University, Shanghai, P.R. China; 3 Department of Radiology, Tangdu Hospital, The Fourth Military Medical University, Xi’an, Shaanxi, P.R. China; Leibniz Institute for Neurobiology, GERMANY

## Abstract

**Purposes:**

Recent neuroimaging studies have shown that people with Internet gaming disorder (IGD) have structural and functional abnormalities in specific brain areas and connections. However, little is known about the alterations of the interhemispheric resting-state functional connectivity (rsFC) in participants with IGD. In the present study, we used a newly developed voxel-mirrored homotopic connectivity (VMHC) method to investigate the interhemispheric rsFC of the whole brain in participants with IGD.

**Methods:**

We compared interhemispheric rsFC between 17 participants with IGD and 24 healthy controls, group-matched on age, gender, and education status. All participants were provided written informed consent. Resting-state functional and structural magnetic resonance images were acquired for all participants. The rsFC between bilateral homotopic voxels was calculated. Regions showing abnormal VMHC in IGD participants were adopted as regions of interest for correlation analyses.

**Results:**

Compared to healthy controls, IGD participants showed decreased VMHC between the left and right superior frontal gyrus (orbital part), inferior frontal gyrus (orbital part), middle frontal gyrus and superior frontal gyrus. Further analyses showed Chen Internet Addiction Scale (CIAS)-related VMHC in superior frontal gyrus (orbital part) and CIAS (r = −0.55, p = 0.02, uncorrected).

**Conclusions:**

Our findings implicate the important role of altered interhemispheric rsFC in the bilateral prefrontal lobe in the neuropathological mechanism of IGD, and provide further supportive evidence for the reclassification of IGD as a behavioral addiction.

## Introduction

Recently, the Internet has become one of the most important academic and recreational tools for adolescents and adults. However, addicted Internet use might lead to negative impacts on the functions of daily life, family relationships, and emotional stability [1]. Internet gaming disorder (IGD), also known as Internet addiction disorder (pathological Internet use), is defined as persistent and recurrent use of the Internet to engage in games, which may lead to significant psychological distress and interfere with daily social life [2]. IGD has become an important global mental health problem, and requires further attention.

Excessive and prolonged Internet gaming can result in negative consequences, such as impaired real-life relationships or academic performance [3]. As it is of significant public health importance, the DSM-V proposed the diagnostic criteria of IGD to define addiction to Internet gaming. It is classified under the conditions for further study of Section III, and it is suggested that more evidence is necessary before being included as a standard disorder in the DSM system. Epidemiological surveys have reported that the prevalence of IGD ranges from 2.4 to 10.7 percent in youth population in China, depending on various different diagnostic criteria [4–6].

Neuroimaging on excessive and addictive use of the Internet is a rapidly growing research field, and has revealed a number of interesting results. These results have both scientific and clinical impacts and help to better understand the neurobiological basis of Internet addiction. Previous researches using various magnetic resonance techniques have reported functional and structural alternations in IGD participants. However, the mechanism of Internet addiction remains unclear. The results of functional magnetic resonance imaging (fMRI) studies indicate that brain regions associated with reward, addiction, craving, and emotion including the nucleus accumbens, amygdala, anterior cingulate, bilateral dorsolateral prefrontal cortex, caudate nucleus, orbitofrontal cortex (OFC), insula, premotor cortices, and precuneus are increasingly activated during game play and presentation of game cues [7–10]. Brand et al. proposed the view that an addictive use of the Internet is linked to functional brain changes involving parts of the prefrontal cortex, accompanied by changes in other cortical (e.g., temporal) and subcortical (e.g., ventral striatum) regions. In addition, there are some hints regarding structural brain changes, which also involve parts of the prefrontal cortex. The functional changes in prefrontal and striatal areas are primarily observable when individuals with Internet addiction perform certain tasks, in particular those measuring executive functions and cue-reactivity[11]. Hong et al. [12] found that adolescents with Internet addiction showed reduced functional connectivity (FC) spanning a distributed network, and the majority of impaired connections involved cortico-subcortical circuits (predominantly prefrontal and parietal cortex). They suggested that IGD is associated with a widespread and significant decrease of functional connectivity in cortico-striatal circuits.

Resting state fMRI (rsfMRI) approaches, which reveal patterns of coherent spontaneous fluctuations in the fMRI signal, offer a means to directly quantify interhemispheric functional interactions. Stark et al. [13] confirmed robustly correlated spontaneous activity between all homotopic regions, which was significantly higher than that between nonhomotopic regions. Although homotopic resting state functional connectivity (rsFC) exhibits regional variation congruent with the brain’s functional hierarchy [14], functional homotopy—the high degree of correlated activity between homotopic interhemispheric counterparts—is still one of the most salient aspects of the brain’s intrinsic functional architecture [15]. Our previous research using resting-state fMRI revealed that reductions in functional connectivity were found in the right inferior temporal gyrus and bilateral parietal cortex. Furthermore, connectivity between the posterior cingulate gyrus and right precuneus, thalamus, caudate, ventral striatum, supplementary motor area, and lingual gyrus was positively correlated with the severity of the problematic behavior in Internet gamers [16].

The present study uses rsfMRI to assess the integrity of interhemispheric interaction in these participants in an attempt to discover whether this new technique can provide additional evidence of the neuropathological mechanism of IGD. The purpose of this study was to examine the interhemispheric synchronous changes of the brain using rsfMRI voxel-mirrored homotopic connectivity (VMHC) in participants with IGD compared with healthy controls. To our best knowledge, this is the first attempt to investigate altered interhemispheric functional connectivity in IDG, based on resting-state fMRI. We hypothesized that IGD subjects would show abnormal inter-hemispheric RSFC. Given evidence for frontal lobe dysfunction associated with IGD, we expected the frontal regions to be particularly affected. We also investigated whether, within patients with IGD, VMHC was related to clinical scores in IGD participants.

## Materials and Methods

### 1. Participants

The participants enrolled and the diagnostic questionnaire and exclusion criteria are described in our previous publication [16]. All participants were recruited from the Department of Child and Adolescent Psychiatry of Shanghai Mental Health Center. They were 14 to 17 years old. We imaged 17 participants whose behaviors matched with the DSM-IV criteria for IGD according to the modified Diagnostic Questionnaire for Internet Addiction (i.e., the YDQ) criteria by Beard [17]. Twenty-four age- and gender-matched healthy individuals with no personal or family history of psychiatric disorders were also imaged as the control group. All participants were right-handed and none of them smoked.

A basic information questionnaire was used to collect demographic information such as gender, age, final year of schooling completed, and hours of Internet use per week. This study was approved by the Ethics Committee of Ren Ji Hospital, School of Medicine, Shanghai Jiao Tong University. The participants and their parents or legal guardians were informed of the aims of our study before the magnetic resonance imaging (MRI) examinations were conducted. Full and written informed consent was obtained from the parents or legal guardians of each participant.

All participants underwent a simple physical examination including blood pressure and heart rate measurements, and were interviewed by a psychiatrist regarding their medical history of nervous, motor, digestive, respiratory, circulation, endocrine, urinary, and reproductive problems. They were then screened for psychiatric disorders with the Mini International Neuropsychiatric Interview for Children and Adolescents (MINI-KID) [18]. The exclusion criteria included a history of substance abuse or dependence, previous hospitalization for psychiatric disorders, or a history of major psychiatric disorders, such as schizophrenia, depression, anxiety disorder, and psychotic episodes. The participants with IGD were not treated with psychotherapy or any medications. The diagnostic questionnaire for IGD was adapted from modified Diagnostic Questionnaire for Internet Addiction (i.e., the YDQ) criteria by Beard [17]. Four questionnaires were used to assess the participants’ clinical features, namely the Chen Internet Addiction Scale (CIAS) [19], the Self-rating Anxiety Scale (SAS) [20], the Self-rating Depression Scale (SDS) [21], and the Barratt Impulsiveness Scale-11 (BIS-11) [22]. The CIAS, developed by Chen, contains 26 items on a four-point Likert scale, and represents the severity of internet addiction. All questionnaires were initially written in English then translated into Chinese.

### 2. MRI acquisition

MRI was conducted using a 3T MRI scanner (GE Signa HDxt 3T, USA). A standard head coil with foam padding was used. During resting-state fMRI, the participants were instructed to keep their eyes closed, remain motionless, stay awake, and not to think of anything in particular. A gradient-echo echo-planar sequence was used in functional imaging. Thirty-four transverse slices (repetition time [TR] = 2000 ms, echo time [TE] = 30 ms, field of view [FOV] = 230 × 230 mm, 3.6 × 3.6 × 4 mm voxel size) aligned along the anterior commissure-posterior commissure line were acquired. Each fMRI scan lasted 440 s. Several other sequences were also acquired, including (1) 3D Fast spoiled Gradient Recalled sequence (3D-FSPGR) images (TR = 6.1 ms, TE = 2.8 ms, TI = 450 ms, slice thickness = 1 mm, gap = 0, flip angle = 15°, FOV = 256 mm × 256 mm, number of slices = 166, 1 × 1 × 1 mm voxel size), (2) axial T1-weighted fast spin-echo sequence (TR = 1725 ms, TE = 24 ms, FOV = 256 × 256 mm, 34 slices, 0.5 × 0.5 × 4 mm voxel size), and (3) axial T2-weighted fast spin-echo sequence (TR = 9000 ms, TE = 120 ms, FOV = 256 × 256 mm, 34 slices, 0.5 × 0.5 × 4 mm voxel size).

### 3. Data analysis

Two-sample t-tests were performed for assessing group comparisons to determine the inter-group demographic differences, and a χ^2^-test was used for gender comparison. A two-tailed p-value of 0.05 was considered statistically significant for all analyses. All statistical analyses were performed using SPSS software (v.19.0.0, IBM, USA). T1- and T2-weighted images were inspected by two experienced neuroradiologists. No gross abnormalities were observed in either group.

Functional images were preprocessed using the Data Processing Assistant for Resting- State Functional (DPARSF3.0 Advanced edition) MR Imaging toolkit [23], which synthesizes procedures in the Resting-State Functional MR imaging toolkit (REST; http://www.restfmri.net) [24], and SPM8 (http://www.fil.ion.ucl.ac.uk/spm). The first 10 images were excluded to ensure steady-state longitudinal magnetization, and the remaining images were then corrected for temporal differences and head motion. After participant selection, neither translation nor rotation parameters in any given data set exceeded ± 1 mm or ± 1°. Moreover, the mean framewise displacement (FD) was computed by averaging FDi from every time point for each subject [25]. There were no differences for the mean FD between groups (p = 0.75). We then co-registered the individual T1 images to functional images. The T1 images were segmented (gray matter, white matter, and cerebrospinal fluid) and normalized to the Montreal Neurologic Institute space using a 12-parameter nonlinear transformation. These transformation parameters were applied to the functional images. To account for differences in the geometric configuration of the cerebral hemispheres, we further transformed the preprocessed functional images to a symmetric space. To achieve this, we used the following procedure: (a) The normalized gray matter images were averaged for all participants to create a mean normalized gray matter image, (b) this image was then averaged with its left-right mirrored version to generate a group-specific symmetrical template, and (c) normalized gray matter images were registered to the symmetric template and applied to the nonlinear transformation to the normalized functional images, and these normalized images finally were resliced at a resolution of 3 × 3 × 3 mm^3^. Finally, we spatially smoothed images with a 6-mm full-width at half-maximum isotropic Gaussian kernel. Before VMHC analysis was performed, several preprocessing steps were taken to remove the sources of possible spurious variance from each voxel’s time series: (a) removing linear trends, (b) regressing out nuisance signals (global mean, white matter, cerebrospinal fluid signals, six head-motion parameters) and spike regressors, and (c) filtering the temporal bandpass (0.01–0.08 Hz).

Finally, we computed Pearson correlations between the time series of every pair of symmetrical interhemispheric voxels. The resulting correlations for each paired voxel constituted a VMHC brain map (Fisher z transformed) and were used for subsequent group-level analyses.

### 4. Statistical Analysis

The significant differences in VMHC between groups were analyzed using a two-sample t test (p < 0.05, corrected with a single voxel height of p < 0.01 and a cluster volume > 486 mm^3^ using a software program [AFNIAlphaSim; http:/afni.nimh.gov/pub/dist/doc/manual/AlphaSim.pdf]) with a gray matter mask produced by the symmetric template outlined previously.

## Results

### 1. Demographics and clinical results

As shown in our previous study [16], 17 IGD participants (four female) were enrolled. The mean age of the IGD participants was 16.94 ± 2.73 years, their mean education was 9.0 ± 2.67 years, and their mean CIAS, SAS, SDS and BIS-11 scores were 64.59 ± 6.43, 45.12 ± 7.41, 50.76 ± 7.93 and 62.53 ± 7.12, respectively. Twenty-four age-, sex- and education-matched control participants (six female) were also examined. Their mean age was 15.87 ± 2.69 years, their mean education was 8.96 ± 2.84 years, and their mean CIAS, SAS, SDS and BIS-11 scores were 45.70 ± 7.81, 42.30 ± 5.34, 47.13 ± 7.31 and 56.25 ± 7.07, respectively. There were no significant differences in the distributions of age, gender, and years of education between the two groups. The participants with IGD showed higher CIAS (p < 0.0001) and BIS-11 (p = 0.01) scores than the controls. No differences in SAS or SDS scores were found between the groups.

### 2. Group differences in VMHC

**Fig 1:**
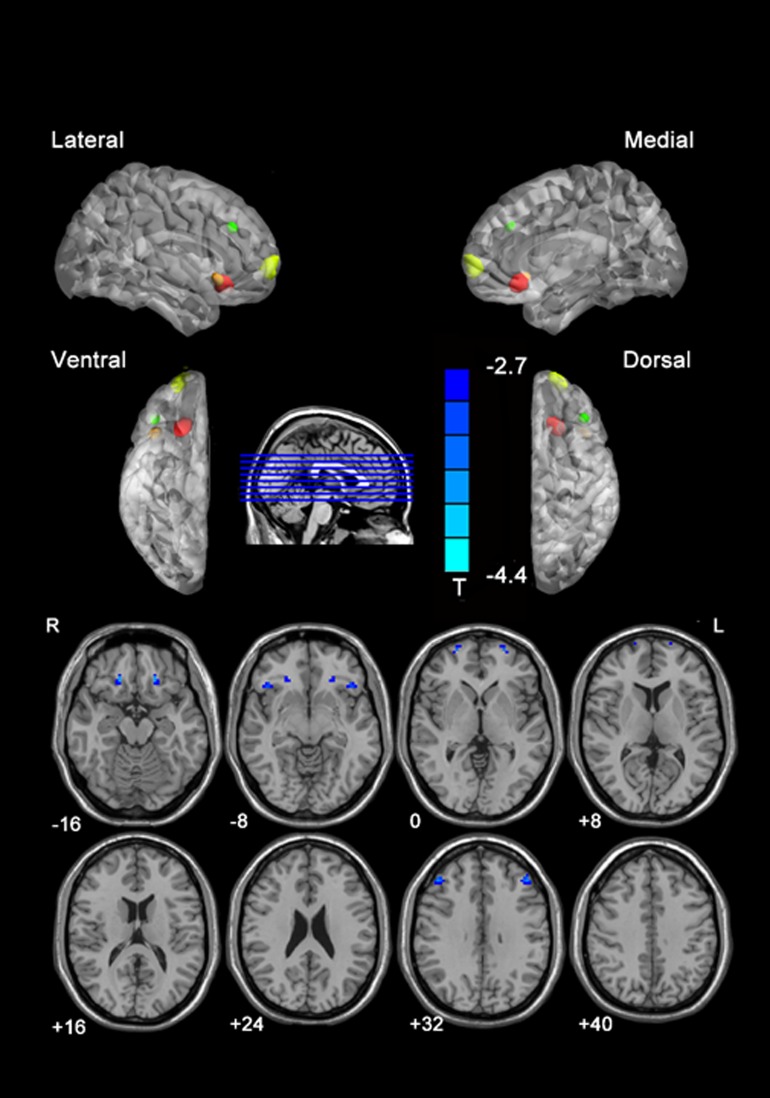
Statistical maps showing VMHC differences between participants with IGD and healthy participants. Blue denotes lower VMHC and the color bars indicate the T value from the t test between groups. In the upper part of the picture, colors represent areas of the brain: red, represents the left and right superior frontal gyrus (orbital part); orange, inferior frontal gyrus (orbital part); yellow, middle frontal gyrus; green, superior frontal gyrus. VMHC, voxel-mirrored homotopic connectivity; IGD, Internet gaming disorder. Note: The left part of the figure (L) represents the participant’s left side, (R) represents the participant’s right side.

Results are shown in Table 1 and Fig. 1. IGD participants showed deficits in VMHC between left and right superior frontal gyrus (orbital part), inferior frontal gyrus (orbital part), middle frontal gyrus (MFG) and superior frontal gyrus (SFG). They did not show greater regional VMHC, as compared with controls.

**Table 1:** Regions showing group differences in voxel-mirrored homotopic connectivity.

	Peak MNI coordinate region	Peak MNI coordinates			Number of cluster voxels	Peak *t* value
		x	y	z		
1	Superior frontal gyrus, orbital part (BA11)	±21	27	−12	18	−4.40
2	Inferior frontal gyrus, orbital part (BA 47)	±45	24	−6	20	−3.57
3	Superior frontal gyrus (BA 10)	±27	63	3	22	−3.95
4	Middle frontal gyrus (BA 45)	±45	36	33	22	−3.76

Abbreviations: MNI, Montreal Neurological Institute; IGA, internet gaming addiction; VMHC, voxel-mirrored homotopic connectivity; BA, Brodmann area.

Note: *t* < 0 indicates smokers with IGA group < HC group in VMHC.

(p < 0.05, AlphaSim-corrected)

### 3. Correlations between VMHC and clinical scores

**Fig 2:**
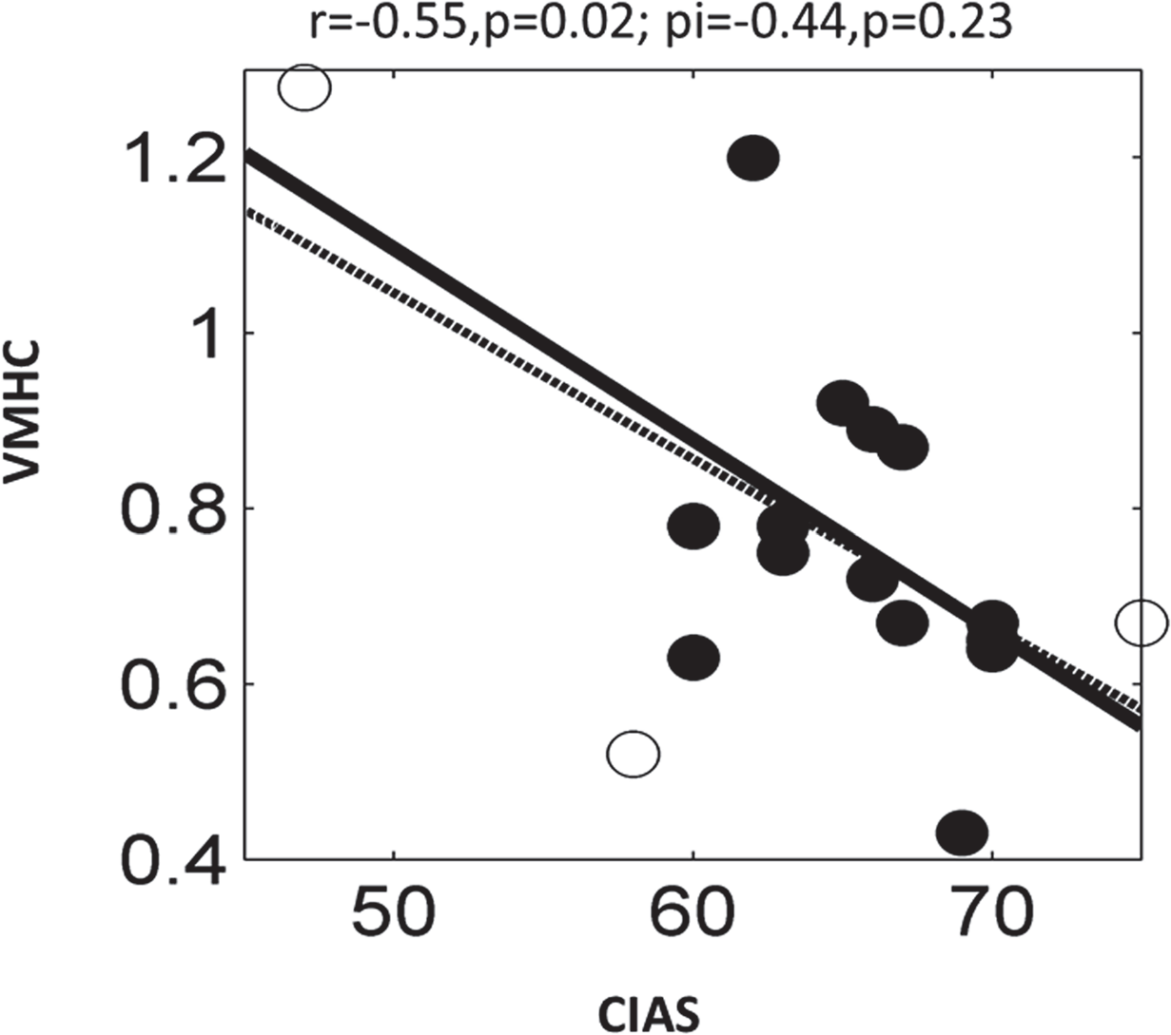
Correlation between VMHC in superior frontal gyrus (orbital part) and CIAS in participants with IGD. Solid and dashed lines represent the best-fit line of the Pearson’s and Shepherd’s correlations, respectively. Outliers are indicated by open circles. VMHC, voxel-mirrored homotopic connectivity; IGD, Internet gaming disorder; CIAS, Chen Internet Addiction Scale.

The mean VMHC values were extracted and averaged within a spherical 4-mm-radius ROI centered on the VMHC group difference peak (reported in Fig. 1 and Table 1). Pearson correlations were performed between VMHC and CIAS, BIS-11, SAS and SDS within the IGD group. To account for the potential influence of outliers, we also used Shepherd’s pi correlation analysis [26]. No significant correlation was found between VMHC and BIS-11, SAS or SDS. Using Pearson’s correlation, a significantly negative correlation was observed between VMHC in superior frontal gyrus (orbital part) and CIAS (r = −0.55, p = 0.02; see Fig. 2) in the IGD group, whereas no significant results were found using Shepherd’s correlation (r = 0.44, p = 0.23) (Fig. 2).

## Discussion

The primary finding of this work is that the correlation between homologous brain regions was reduced in participants with IGD. These reductions were observed in the prefrontal lobe, including superior frontal gyrus (orbital part), inferior frontal gyrus (orbital part), SFG and MFC. The robust homotopic functional connectivity between the two hemispheres has been demonstrated to be a salient characteristic of the brain’s intrinsic functional architecture [13, 27], likely reflecting the important role of interhemispheric communication in the brain’s functional integrity.

The mechanism underlying these deficits in VMHC is still unknown. As Hoptman et al.[28] stated, they could be related to the widespread white matter integrity abnormalities [29]; deficits in white matter connectivity in the corpus callosum could disrupt the synchrony between homotopically connected regions because neural signals are not transmitted with fidelity [30, 31]. Dysfunctions in local gray matter structure might account for these deficits [28] because reduced neuropil or aberrant local oscillatory firing within gray matter structure may disrupt coherent low frequency oscillatory activity and/or its generation in one region, and thereby impair its functional connectivity with other regions. Finally, alternative pathways (e.g., subcortical) exist, although the callosum is the largest conduit for information transfer and coordination between the hemispheres. However, previous research revealed that IGD participants had significantly lower FA than controls in the bilateral genu and body of the corpus callosum [32, 33]. The actual explanations account for the present findings awaits combined functional/structural studies.

The OFC is a prefrontal cortex region in the frontal lobes that consists of the association cortex areas Brodmann areas 10, 11 and 47 [34]. It is a region involved in processing emotions, and participates in craving, maladaptive decision-making processes, and engagement in compulsive behaviors, each of which is integral to addiction [35]. The OFC is thought to contribute to goal-directed behavior through the assessment of motivationally significant stimuli and the selection of behavior to yield desired outcomes [36]. Its extensive connections with the striatum and limbic system reveal that it integrates emotion and natural drive from limbic and subcortical areas to assess the reward value against previous experience [37, 38]. It creates and maintains expectations about possible reward related to reinforcement [39]. Previous research has reported decreased cortical thickness in the left OFC [40] and lower FA in OFC white matter [32]. The voxel-based morphometry (VBM) method has revealed gray matter deficits in the OFC in IGD individuals [33]. In the current study, a significant negative correlation was observed between VMHC in superior frontal gyrus (orbital part) and CIAS, indicating that the OFC plays an important role in the neuropathological mechanism of IGD.

Superior frontal gyrus (SFG) is associated with higher craving experience. Leung [41] suggested that game designers try to satisfy a players’ sense of achievement and encourage their desire to pursue continuing victories while playing. Thus, internet gaming behavior can be persistently rewarded by feelings of being in control, the synchronous interactive quality, immediate achievement, and the freedom of self-representation. This view has been supported in subsequent research. Dong et al. found that. IGD participants showed higher SFG activation after continuous wins than healthy controls [9]. A study on nicotine addiction samples found that significantly higher activations in the SFG were observed in response to smoking cues than to neutral cues [42]. As for MFG, a similar result was reported for inhibitory errors. Heitzeg et al. suggested that blunted left MFG activation during performance errors may underlie problems in adapting behavior appropriately, leading to under-controlled behavior [43]. A behavioral consequence of blunted activity in MFG may be difficulty in adjusting one’s own behavior appropriately.

Until now, no definite consensus has yet been reached on the neuropathological mechanism of IGD. Some researchers have suggested that it should be classified as an impulse control disorder considering impulsivity as a common trait and vulnerability markers of IGD [17]. Others have argued that IGD should be included in behavioral addictions because of the impairment in behavioral control, craving and diminished recognition of significant problems with one’s behavior and interpersonal relationships, as defined by the American Society of Addiction Medicine [44]. An alternative theoretical model of behavior addiction addressing the involvement of both brain reward pathways (the ventral striatum) and the regulatory system (the prefrontal cortex) has also been raised [9, 45]. We found that the deficits in VMHC of the prefrontal lobe including OFC, SFC and MFC that relate the functions of decision-making, craving and inhibitory errors share the same neural mechanism as this model.

In addition to the relatively small sample size, other limitations of our study should be noted. Firstly, we applied a symmetrical standard template and smoothed the imaging data to improve the functional correlations between mirrored regions. However, the human brain is not strictly symmetrical. Although morphometric asymmetry cannot account for the reduced VMHC [46], the effects of methodological symmetry cannot be completely eliminated. Secondly, gray matter volume and white matter diffusion are not assessed in the current study. Thus, there is no way to determine the relationship between VMHC and gray matter volume or white matter diffusion. Thirdly, this study focused on the internet gaming sub-group of IA, but no direct comparisons were made with other IA subgroups; therefore, it remains to be investigated how well the results may be translated to other IA subgroups, if at all.

Despite the limitations, the current study found that participants with IGD showed deficits in VMHC between bilateral SFG (orbital part), MFG and SFG compared to healthy controls. Furthermore, CIAS negatively correlated with the reduction in VMHC in left and right SFG (orbital part) (Pearson’s correlation). These findings implicate the important role of altered interhemispheric rsFC in the prefrontal lobe in the neuropathological mechanism of IGD. Considering the overlapping role of the prefrontal lobe in the reward and self-regulatory system, our results provide further supportive evidence for the reclassification of IGD as a behavioral addiction.
